# Characterisation of the Filler Fraction in CAD/CAM Resin-Based Composites

**DOI:** 10.3390/ma14081986

**Published:** 2021-04-15

**Authors:** Andreas Koenig, Julius Schmidtke, Leonie Schmohl, Sibylle Schneider-Feyrer, Martin Rosentritt, Hieronymus Hoelzig, Gert Kloess, Ketpat Vejjasilpa, Michaela Schulz-Siegmund, Florian Fuchs, Sebastian Hahnel

**Affiliations:** 1 Department of Dental Prosthetics and Materials Science, Leipzig University, 04103 Leipzig, Germany; julius.schmidtke@medizin.uni-leipzig.de (J.S.); leonie.schmohl@medizin.uni-leipzig.de (L.S.); florian.fuchs@medizin.uni-leipzig.de (F.F.); sebastian.hahnel@medizin.uni-leipzig.de (S.H.); 2 Department of Prosthetic Dentistry, Regensburg University Medical Centre, 93042 Regensburg, Germany; sibylle.schneider-feyrer@klinik.uni-regensburg.de (S.S.-F.); martin.rosentritt@klinik.uni-regensburg.de (M.R.); 3 Institute of Mineralogy, Crystallography and Materials Science, Leipzig University, 04275 Leipzig, Germany; hieronymus.hoelzig@uni-leipzig.de (H.H.); kloess@uni-leipzig.de (G.K.); 4 Institute of Pharmacy, Pharmaceutical Technology, Leipzig University, 04317 Leipzig, Germany; kv38gaxo@studserv.uni-leipzig.de (K.V.); schulz@uni-leipzig.de (M.S.-S.)

**Keywords:** RBC, sphericity, micro-X-ray computer tomography, dental glasses, microstructure, direct composites, filler size distribution, microtomography, X-ray diffraction

## Abstract

The performance of dental resin-based composites (RBCs) heavily depends on the characteristic properties of the individual filler fraction. As specific information regarding the properties of the filler fraction is often missing, the current study aims to characterize the filler fractions of several contemporary computer-aided design/computer-aided manufacturing (CAD/CAM) RBCs from a material science point of view. The filler fractions of seven commercially available CAD/CAM RBCs featuring different translucency variants were analysed using Scanning Electron Microscopy (SEM) with Energy Dispersive X-ray Spectroscopy (EDS), Micro-X-ray Computed Tomography (µXCT), Thermogravimetric Analysis (TG) and X-ray Diffractometry (XRD). All CAD/CAM RBCs investigated included midifill hybrid type filler fractions, and the size of the individual particles was clearly larger than the individual specifications of the manufacturer. The fillers in Shofu Block HC featured a sphericity of ≈0.8, while it was <0.7 in all other RBCs. All RBCs featured only X-ray amorphous phases. However, in Lava Ultimate, zircon crystals with low crystallinity were detected. In some CAD/CAM RBCs, inhomogeneities (X-ray opaque fillers or pores) with a size <80 µm were identified, but the effects were minor in relation to the total volume (<0.01 vol.%). The characteristic parameters of the filler fraction in RBCs are essential for the interpretation of the individual material’s mechanical and optical properties.

## 1. Introduction

Dental resin-based composites (RBCs) include a group of tooth-coloured, nonmetallic restorative materials, which consist of a polymer resin matrix supplemented with a dispersed inorganic filler fraction.

Unlike thermoplastics, the polymer matrix (duroplastic) features a close-meshed rather than a chain structure. The polymers consist of dimethacrylate (DMA) monomers, which include two terminal methacrylate groups with a variable middle section. Bisphenol A-glycidyl methacrylate (Bis-GMA) in combination with aliphatic co-monomers, such as triethylene glycol dimethacrylate (TEGDMA) and ethylene glycol dimethacrylate (EGDMA) as well as urethane dimethacrylate (UDMA), are currently used undilutedly or in combination with other DMA monomers [1].

The inorganic filler fraction supplemented to the monomers include filler particles with various sizes, contents, and compositions. Judging from the literature as well as the technical information issued by the individual manufacturer, fillers may be fabricated from glass ceramics or alumina, silica, quartz, and yttrium fluoride particles, zirconia/silica clusters, strontium glass, barium aluminium fluoride glass, or fumed silica. In many cases, the manufacturers do not provide any information on the type of filler at all (see Table 1). The filler particles are coated with silanes to ensure chemical bonding between the organic and inorganic compounds of the RBC [2,3]. Randolph et al. [4] distinguishes hybrid filler particles with sizes ≤6 µm and nanohybrid filler particles with a size ≤1 µm. Additionally, Ferracane [5] distinguishes nanofill (5–100 nm), minifill (0.6–1 µm + 40 nm), and midifill (1–10 µm + 40 nm) filler particles. The volume content of the filler fraction can be categorized into ultralow-fill (<50 wt%), low-fill (50–74 wt%), and compact (>74 wt%) [4].

Dental RBCs may be further subgrouped into direct (intraorally polymerized) and indirect (extraoral polymerized) materials. Indirect RBCs are also known as computer-aided design/computer-aided manufacturing (CAD/CAM) RBCs and are polymerized under industrial conditions and supplied as blocks or discs. Subsequent to CAD [2], the restoration is milled from the blocks or discs (CAM) without any final polymerization or sintering process [3].

The conventional viewpoint is that CAD/CAM RBCs feature improved properties in comparison to direct RBCs or filler-supplemented poly methyl methacrylate (PMMA) polymers, which can be attributed to the following:

- Industrial polymerization under high temperatures and pressure. With regard to this aspect, Nguyen et al. showed that a polymerization temperature of 180 °C and pressure of 250 mPa for 60 min result in ”a significant […] increase in flexural strength, hardness, and density” of RBC blocks [6]. The conversion rate increases to over 90% [7].

Several studies document that the brittleness, hardness, and elastic modulus of CAD/CAM RBCs are lower in comparison to ceramics and closer to the properties of enamel and dentine [1,8,9]. The biaxial flexural strength of RBC crowns has been reported to be lower than for crowns fabricated from lithium disilicate ceramic, yet the fracture load of indirect RBC crowns (3.3 to 3.9 kN) is almost similar to those fabricated from lithium disilicate-ceramic (IPS e.max CAD, 3.3 kN). These values exceed the average bite force measured in molar teeth (700–900 N) by far [10], which can be explained with the RBCs’ low sensitivity against small damages on the surface in comparison to ceramics [11], their higher Weibull modulus (10–17 for composites vs. 8 for IPS e.max CAD) [12], and their high fracture energy.

RBCs show a lower abrasion wear than those that are manually polymerized, and both have higher wear values than glass-ceramic [13,14]. The laboratory results were confirmed in a clinical study [15]. Lithium disilicate ceramics (glass-ceramics) showed more stable wear behaviour after two years compared to composites. At the same time, the direct and indirect composites produce less abrasive wear of antagonistic teeth than the glass-ceramic [13,14,15,16]. The wear behaviour of RBC can be explained by the high surface hardness of the fillers and the elastic modulus compared to glass-ceramic.

While filler composition and characteristics and their impact on the performance of direct RBCs have been extensively analysed in recent years, only few studies addressing the filler fraction in CAD/CAM RBCs are available.

In direct RBCs, the size of the filler particles employed has continuously decreased to 1 µm [5] and even smaller nanoparticles (<100 nm) [17]. Modern direct nanohybrid RBC formulations predominantly include both submicron (<1 µm) and nanoparticles (<100 nm) to reduce interparticle spacing, increase filler content, and improve mechanical properties [18]. Several studies on direct composites show that with increasing filler content, the volumetric shrinkage decreases and surface hardness increases. With decreasing filler size (from hybrid or microhybrid to nanohybrid), the microhardness (Vickers), in particular, can be additionally increased [19,20,21,22]. Hahnel et al. showed on experimental RBCs that even small changes in filler size (Ø 1.5/2.5 µm) and content (87–91 wt%) lead to significant changes in mechanical properties (flexural strength and hardness) [23]. Filler morphology and composition also influence the mechanical properties of direct RBCs, although no general trend can be observed. Randolph et al. [4] emphasize the difficulty of identifying a correlation between a single filler variable and a distinct mechanical property by using commercial materials where many filler variables differ simultaneously. With regard to the optical properties of am RBC, the effects of filler content, filler shape, filler size, and composition of the filler fraction have been reported, affecting translucency [24,25,26] and opacity [27].

Hussain et al. investigated the filler content of three CAD/CAM RBCs in comparison to two direct RBCs by thermogravimetric analysis and filler morphology by scanning electron microscopy (SEM) in combination with energy dispersive X-ray spectroscopy (EDS) for elemental analysis [28]. Although the study aimed to evaluate differences between the mechanical properties (hardness) and filler particles between direct and indirect RBCs, significant differences between the various CAD/CAM RBCs regarding filler content, filler composition, filler size, distribution of the filler particles, and hardness are obvious. Moreover, the authors highlighted that there is a discrepancy between filler content issued by the manufacturer and the filler content identified in their study [28]. Other studies investigating CAD/CAM RBCs report correlations among filler weight and microhardness, elastic modulus, or creep [3,9,10,16,29,30]. The fracture load of molar crowns fabricated from nanofiller RBCs was higher in comparison to those fabricated from RBCs with large spherical fillers (Shofu HC) [10,31].

To date, there is only limited scientific literature available that investigates correlations between the properties of the filler fraction in CAD/CAM RBCs and their individual mechanical and optical properties, which is particularly surprising, as most indirect RBCs are available in different colours and translucency grades. Thus, the aim of the current study was to characterize the filler fraction of various CAD/CAM RBCs with either high or low translucency but identical colour. The null hypothesis of the current study was that CAD/CAM RBCs include nanofillers based on glass and feature a homogeneous structure without defects as a result of industrial polymerization.

## 2. Materials and Methods

### 2.1. Materials

Various popular commercially available CAD/CAM resin-based composite (RBC) blocks (colour A2) were selected for analysis in the current study. To compare material characteristics dependent on translucency, low/medium- and high-translucency variants were analysed for each material (LuxaCam Composite (DMG) was only available in a single translucency). The product information provided by the manufacturers is displayed in Table 1.

**Table 1:** Information on composition and weight of the CAD/CAM resin-based composite (RBC) blocks analysed in the current study as specified by the manufacturer; mechanical properties based on manufacturer and Rosentritt et al. [32].

Material	Code	Manufacturer	Variants	LOT	E-Modulus GPa Flexural Strength MPa	Composition		Filler Weight wt%
						Organic	Inorganic	
BRILLIANT Crios	CBC	COLTENE Holding AG, Altstätten, Switzerland	A2 HT 14	I44747	10 198	cross-linked methacrylates	barium glass (<1.0 µm) amorphous silica (<20 nm)	70.7
			A2 LT 14	IO3O77				
Cerasmart^TM^	GCC	GC Corporation, Tokyo, Japan	A2 HT 14L	1809051	10 246	-	-	-
			A2 LT 14L	1710041				
Lava^TM^ Ultimate	3LU	3M Deutschland GmbH, Neuss, Germany	A2 HT 14L	N987419		-	silica nanomers (20 nm) zirconia nanomers (4–11 nm) zirconia–silicia nanoclusters (0.6–10 µm)	app. 80
			A2 LT 14L	N401476	12–15 170–210			
Shofu Block HC	SB	Shofu Dental GmbH, Ratingen, Germany	A2 HT M	071601	8–10 170–190	-	-	-
			A2 LT M	0818225				
Tetric^®^ CAD	TC	Ivoclar Vivadent GmbH, Ellwangen, Germany	A2 HT C14	W90501		cross-linked dimethacrylate (Bis-GMA, Bis-EMA, TEGDMA, UDMA)	barium aluminium silicate glass (<1 µm) silicon dioxide (<20 nm)	71.1
			A2 MT C14	Y50470	10 272			
Grandio^®^ blocs	VGB	VOCO GmbH, Cuxhaven, Germany	A2 HT 14L	1831230	15–18 250–290	-	-	86
			A2 LT 14L	1842286				
LuxaCAM Composite	LC	DMG GmbH, Hamburg, Germany	A2 14L	795497	-	highly networked polymer	silicate glass filler	app. 70

### 2.2. Methods

#### 2.2.1. Thermogravimetric Analysis

Thermogravimetric analysis (TG) was performed to determine the polymer and filler content (mass percent) of the various CAD/CAM RBCs using the TGA/DSC1 STAReSystems with STARe Software 14.0 (Mettler Toledo, Columbus, OH, USA). Cylindrical specimens (Ø/h = 4/1 mm) were milled from the various CAD/CAM RBCs using a five-axis milling machine (Sirona inLab MC XL, Dentsply Sirona, Bensheim, Germany). A single specimen of each material (approx. 13 mg) was placed in an open melting pot made of Al_2_O_3_ (THEPRO GbR, Heinsberg, Germany) and heated under nitrogen atmosphere (nitrogen flow rate 40 mL/min) as follows: heating from 25 to 100 °C with 10 K/min, 20 min isothermal, further heating to 900 °C with 10 K/min, 20 min isothermal.

The results of the measurements were evaluated using STARe Software 14.0 (Mettler Toledo, Columbus, OH, USA) and OriginPro 2017G (OriginLab Corporation, Northampton, MA, USA). Noise reduction in derivative thermogravimetric curves was performed using either fast Fourier transformation (FFT) and low-pass filtering or the Savitzky–Golay method, where appropriate. Relative mass values were calculated, normalizing the residual mass after heating to 100 °C to 100%. In order to estimate the reproducibility, TG measurements were carried out on five subsamples of BRILLIANT Crios (CBC). The standard deviation was 0.8%.

#### 2.2.2. Scanning Electron Microscopy (SEM) with Energy Dispersive X-ray Spectroscopy (EDS)

SEM was performed to investigate filler size distribution and morphology. Each CAD/CAM RBC was cut into rectangular bars of 3 mm thickness using the IsoMet^®^ 4000 Linear Precision saw (Buehler Ltd., Lake Bluff, IL, USA) and a diamond cut-off wheel (M4D18, Struers GmbH, Willich, Germany) under constant water irrigation (ISO 6872:2019-01). Subsequently, all specimens were polished (Pedemin-2, Struers GmbH, Willich, Germany) by successive metallographic papers (Table 2). Quality of polishing was verified using a laser scanning microscope (VK-X, Keyence Deutschland GmbH, Neu-Isenburg, Germany). Surface quality was rated sufficient when Sa ≤ 0.01 µm was achieved in ten different and randomly selected locations on the surface of each sample. Finally, the specimens were cleaned in an ultrasonic bath (BANDELIN electronic GmbH & Co. KG, Berlin, Germany) with distilled water for 10 min and stored under dry conditions at room temperature for 24 h.

The specimens were mounted on aluminium stubs, coated with a conductive varnish and examined using Quanta 400 FEG (FEI Company, Hillsboro, OR, USA) with magnifications of 10,000, 40,000, and 50,000 at 7 kV in Back-Scattered Electron (BSE) mode under low vacuum. The distribution of filler size and filler morphology was determined at a magnification of 10,000. The analyses were based on the different grey values using ImageJ (National Institutes of Health, 1.51d, Bethesda, MD, USA). Points of contact between individual particles were manually separated before automatic segmentation, which was applied on the turning point of the radial grey value profile (logistician of single fillers in accordance with [33]). The distribution of filler size and the sphericity of the filler particles were quantified on single pictures based on Bresenham’s circle algorithm, which was invented in 1962 by Jack E. Bresenham from IBM [34]. Depending on the filler size, which was analysed in terms of size and sphericity, the number of analysed fillers was between 300 (SB) and 5100 (GCC) in each SEM picture.

In order to determine the chemical composition of the fillers, an energy dispersive X-ray spectroscopy (EDS) system with the designation EDAX, APEX EDS Analysis System, Octane Elect (AMETEK Inc., Berwyn, PA, USA) was performed on different regions per cross section.

#### 2.2.3. X-ray Diffractometry (XRD)

For XRD, the CAD/CAM RBCs were cut into 3 mm-thick pieces and polished in the same way as for the SEM investigations. The analyses were performed using a D8 Discover (Bruker AXS, Karlsruhe, Germany) X-ray diffractometer equipped with a VÅNTEC-500 area detector to characterize the crystalline phase. Cu Kα radiation (λ = 1.5418 Å) and an X-ray setting with 40 kV and 40 mA were used.

#### 2.2.4. Micro-X-ray Computed Tomography (µXCT)

Micro-X-Ray computer tomography (µXCT) was used to identify local discontinuities in specimens. The μXCT (FhG e.V., Dresden, Germany) was performed with an X-ray tube FXE 225.99 (focal spot diameter 0.6 µm, tungsten target; YXLON International GmbH, Hamburg, Germany) and a 2D-detector 1621 × N (2048 × 2048 pitches, CsI, pitch size 200^2^ µm^2^; PerkinElmer Inc., Waltham, MA, USA). Since the experimental effort required for such measurements is very high, no major differences between the translucencies are to be assumed with other methods. For this reason, only one sample per material was investigated.

The microfocus transmission mode was used to identify local discontinuities with low density, such as pores, air voids or microcracks, and polymer agglomerates, as well as those with high density, such as filler agglomerates. One cylindrical specimen (Ø/h = 2/10 mm) per material was milled from the various CAD/CAM RBCs similar to thermal analysis. Depending on the radiopacity of each material, the X-ray power was varied between 20 and 27 watts (beam energy 150/180/200 kV and flux 150 μA) using a copper filter (0.5 mm) and a step size of 0.45/360° (800 positions). The voxel edge length as an indicator for maximum resolution was 1.8 μm (V = 5.8 μm^3^). The grey value-specific three-dimensional data sets were cut and orientated with ImageJ. To quantify the discontinuities, a region of interest (ROI) with the dimension (Ø/h = 1.20/1.77 mm) was used for the analyses with VGStudioMax (version 2.0, Volume Graphics GmbH, Heidelberg, Germany). The threshold was determined for the largest discontinuities based on the grey value distribution on the transition zone [33] for normal and inverse ROI with ImageJ. Using the defection tool in VGStudioMax, the size and number of the discontinuities were determined. Only discontinuities in excess of 1000 µm^3^ (≈188 voxels) were determined.

## 3. Results

### 3.1. Microstructure

**Figure 1:**
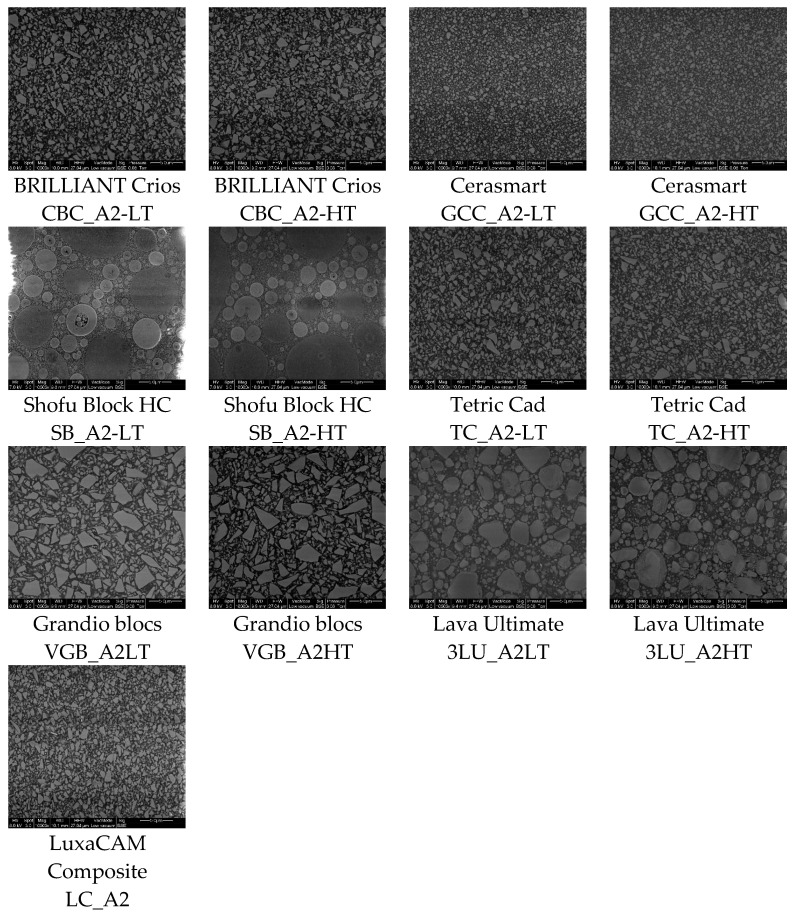
Scanning electron microscopy (SEM) images (25 × 23.5 µm^2^) of cross sections of the various CAD/CAM RBCs with a constant magnification of 10,000 in BSE mode.

The microstructure of the CAD/CAM RBCs is characterized by many inorganic fillers isolated from one another with different sizes and sphericities, as well as by a connecting an organic matrix (Figure 1).

**Figure 2:**
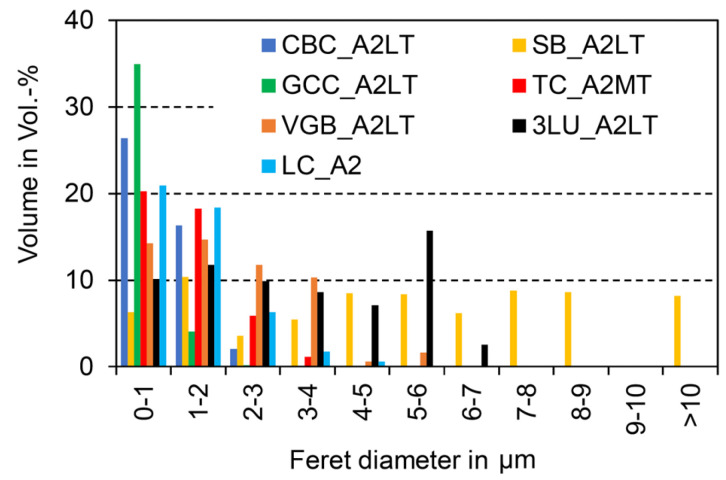
Filler size distribution (selection; see Supplementary Materials for overall results) based on one SEM picture with max. 5100 single particles.

**Figure 3:**
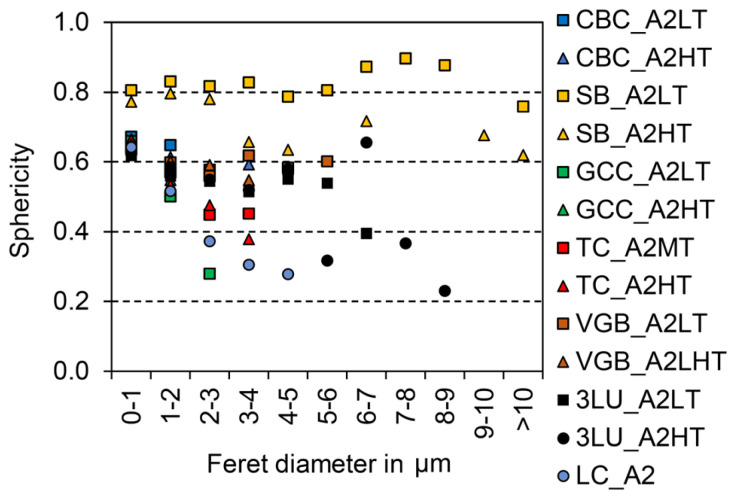
Filler sphericity distribution (1.0 indicates the cross section of the filler is a circle) based on one SEM picture with max. 5100 single particles.

The largest filler particles were identified in Shofu Block HC (SB), followed by Lava Ultimate (3LU) and Grandio blocs (VGB). Taking into account that the largest dimension of a filler particle is statistically unoccupied and that the size distribution is dependent on the resolution (pixel edge length ~5.3 nm), filler sizes between ~100 nm (measured manually with magnification of 50,000 for Cerasmart (GCC) and BRILLIANT Crios (CBC)) and 12 µm (SB) could be detected by SEM (Figure 2). The fillers in SB show a high sphericity (approximately ≈ 0.8). In the other composites, more sharp-edged filler shapes with a sphericity lower than 0.7 were detected (Figure 3).

The CAD/CAM RBCs featured filler proportions between 37.2 Vol.-%/61.6 wt% and 74.4 Vol.-%/83.1 wt%. Taking measurement uncertainties into account, the differences between high-translucency (HT) and low-translucency (LT) variants regarding filler mass and volume proportions were negligible. For all materials, measured values were slightly below the specifications indicated by the manufacturers (Table 3). The thermogravimetric analysis (TG) charts with specific mass loss stages are supplied as Supplementary Materials.

**Figure 4:**
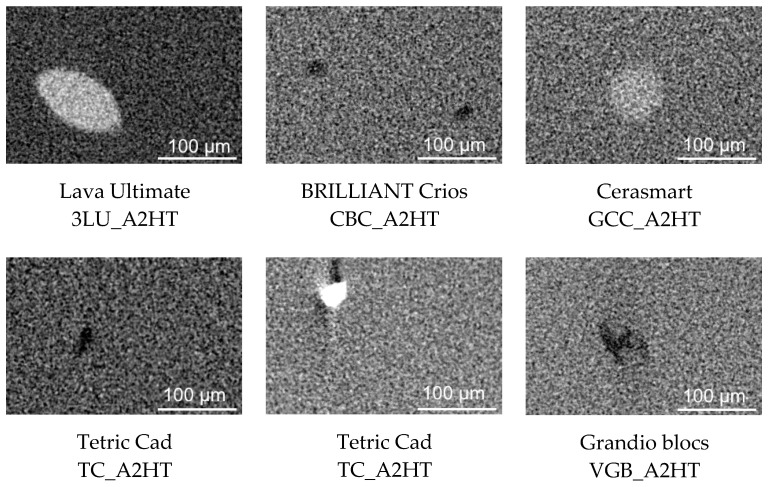
Cross sections of local discontinuities identified by µXCT in CAD/CAM RBCs (HT).

Using high-resolution 3D data sets, micro-X-ray computed tomography (µXCT) detected the least inhomogeneity in the CAD/CAM RBCs. In discontinuities with low density, a clear distinction between the polymer and gas is non-negligible, while local areas with high density can be assigned to fillers with X-ray contrast tracers. The largest discontinuities identified for each CAD/CAM RBCs are visualized in Figure 4 and quantified in Table 4. No discontinuities were identified for CAD/CAM RBCs that are not displayed in Table 4.

### 3.2. Chemical and Mineralogical Phase Composition

Assuming that in optimal measurement conditions the grey value of a pixel is directly related to atomic mass, locations in the filler were selected by SEM, from which point analyses were performed by EDS. For this reason, different ranges were examined for the various CAD/CAM RBCs (Table 5). All fillers were based on silicon (Si) and oxygen (O). In the case of 3LU and SB, zirconium (Zr) was identified, while in all other materials aluminium (Al) was detected. Barium (Ba) was determined in CBC, GCC, Tetric Cad (TC), and VGB.

**Figure 5:**
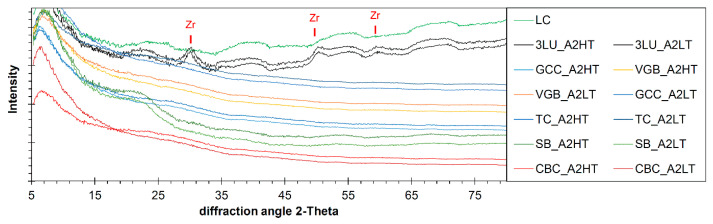
XRD patterns of the filler particles in the various CAD/CAM RBCs (3LU-Lava Ultimate, CBC-BRILLIANT Crios, GCC-Cerasmart, LC-LuxaCAM Composite, SB-Shofu Block HC, TC-Tetric Cad, VGB-Grandio blocs); only in 3LU samples crystalline phases could be detected—in this case zirconia (Zr).

With the exception of 3LU, X-ray diffractometry (XRD) identified that all CAD/CAM RBCS featured only X-ray amorphous phases. Almost no differences were detected between high-translucency (HT) and low-translucency (LT) samples. For Luxa-CAM Composite (LC), a similar phase inventory was identified as for 3LU. Only the area around 30 theta shows a reflex with a larger half-value width, which can be assigned to barely crystalline zirconia (Figure 5).

## 4. Discussion

The null hypothesis of the current study, stating that CAD/CAM RBCs include nano-fillers based on glass and feature a homogeneous structure without defects as a result from industrial polymerization, was rejected (Table 6).

Unlike polymer-infiltrated ceramics (PIC), such as VITA Enamic (H. Rauter GmbH & Co. KG, Bad Säckingen, Germany), which feature a ceramic-polymer network structure, the filler fractions in CAD/CAM RBCs lie isolated in the polymer matrix. In order to produce materials with high packing density, filler fractions with grain bands rather than individual grain sizes were used. A large grain band coincides with high packing density and minimized polymer content. Limiting the maximum grain size is important for homogeneous material behaviour (aesthetic properties, mechanical behaviour), but also especially after mechanical stress in the form of abrasion, when individual local parts (e.g., fillers) are mobilised on the composite surface. The filler size distribution of the CAD/CAM RBCs investigated ranges from the lower nm range to a maximum size of approximately 12 µm (SB) (Figure 2 and Figure 3), and for all materials, filler sizes were clearly larger than those issued by the manufacturers (Table 1). Based on the categorization of [4,5], the filler distribution of the CAD/CAM RBCs investigated can be classified as midifill hybrid rather than nanohybrid. Due to the large grain bands, a higher packing density can occur.

**Figure 6:**
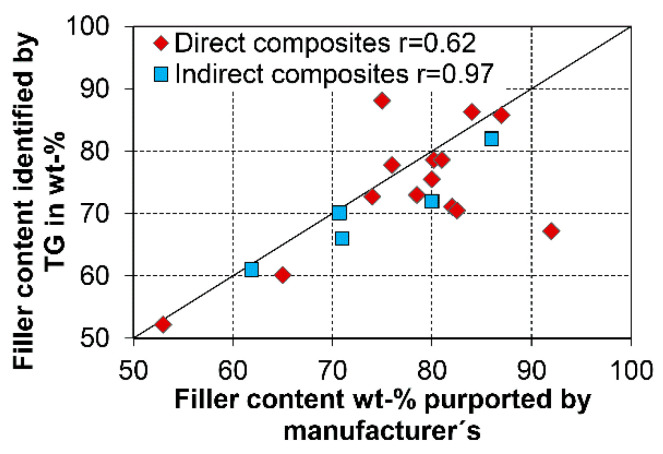
Filler content as identified by TG in the current study and data supplied by the manufacturers for direct (Randolph et al. 2016 [4]) and indirect RBCs (Table 3).

**Figure 7:**
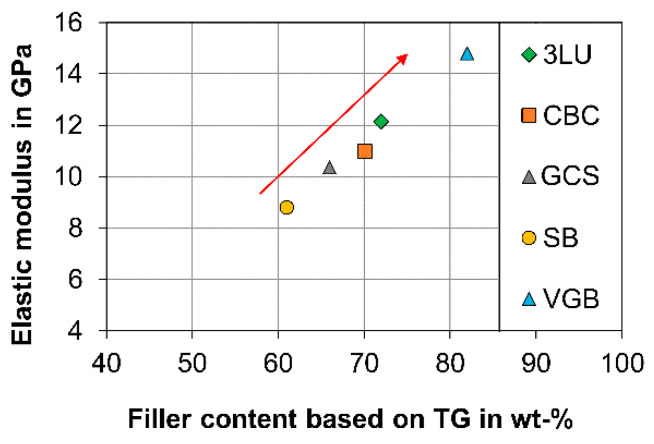
Filler content as identified by TG in correlation with the elastic modulus (manufacturer’s data) for indirect RBCs (3LU-Lava Ultimate, CBC-BRILLIANT Crios, GCS-Cerasmart, SB-Shofu Block HC, VGB-Grandio blocs).

Similar to filler size, the filler content as determined by thermogravimetric analysis (TG) in the current study was lower than issued by the manufacturer (Table 3), which is a phenomenon that was observed for all investigated CAD/CAM RBCs. Silanol condensation during the pyrolysis of the polymer matrix and the remaining carbon due to the inert gas might serve as explanations higher filler content. In the same way the condensation of the silanol (Si-O-H) in the range of 600 °C lead to a decrease the filler content. In comparison to the filler content of direct RBCs, the filler content of CAD/CAM RBCs is regularly higher (Figure 6). The conventional viewpoint is that the mechanical properties, such as the modulus of elasticity (Figure 7), the flexural strength [32,35], the wear resistance [13,36,37], or the hardness [8], increase with filler content. At the same time, small filler sizes and lower filler content increase light transmittance [24,25]. Moreover, wear properties steadily approach [37] the characteristics of the filler particles when the filler content is increased. The linear relationship between filler content (V_polymer_, V_Filler_) and the modulus of elasticity (E_Polymer_, E_Filler_, E_Composite_) is consistent with the simple Voigt’s model, which is most frequently applied for fibre-reinforced composites (formula 1). The linear relationship is an indicator of efficient bonding between the resin matrix and filler particles, a high degree of polymerization, and a homogeneous particle distribution, as well as a few pores in the matrix.

(1) $$E_{\mathrm{composite}}=V_{\mathrm{Polymer}}E_{\mathrm{Polymer}}+V_{\mathrm{Filler}}E_{\mathrm{Filler}}$$

**Figure 8:**
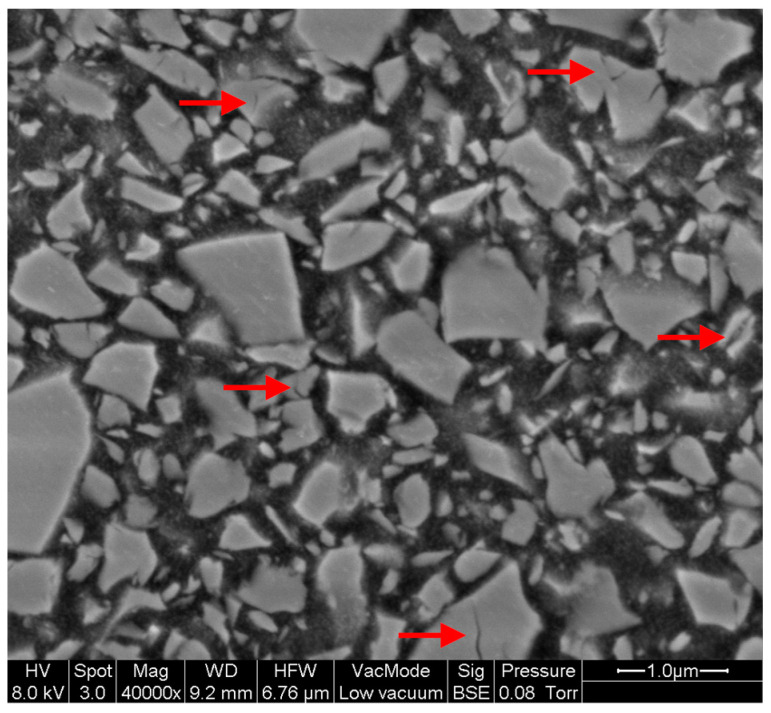
Example for microcracks (red arrows) observed in the filler particles in BRILLIANT Crios (CBC_A2HT).

Apart from filler size, differences in grain shape or sphericity were identified between the various CAD/CAM RBCs, which allows us to draw conclusions on the production of the glasses and on the influence of the filler particles on the mode of action of RBCs. For example, low sphericity indicates mechanical crushing of larger particles. Spherical filler particles were only detected in SB. It is likely that the fillers were not mechanically stressed and were produced by other processes, such as a pyrogenic process (fumed silica) or chemical sol–gel process [38,39]. Under high magnifications (50,000), cracks (Figure 8, red arrows) could be sporadically identified in the fillers of CBC, LC, and VGB, which might be a result of mechanical crushing.

It is well known that with increasing sphericity, the specific surface referring to the volume of the particles decreases. As a result, the mechanical bonding between filler particles and the polymer matrix may improve with a lower sphericity. The colour differences increase with increasing filler content and specific surface area (irregular and smaller fillers) [24]. Irregularly shaped filler particles, as identified in GCC, TC, and VGB, shift the colour of the RBC to a more green and yellow spectrum [24], which is why the dye in the materials must be modified accordingly.

**Figure 9:**
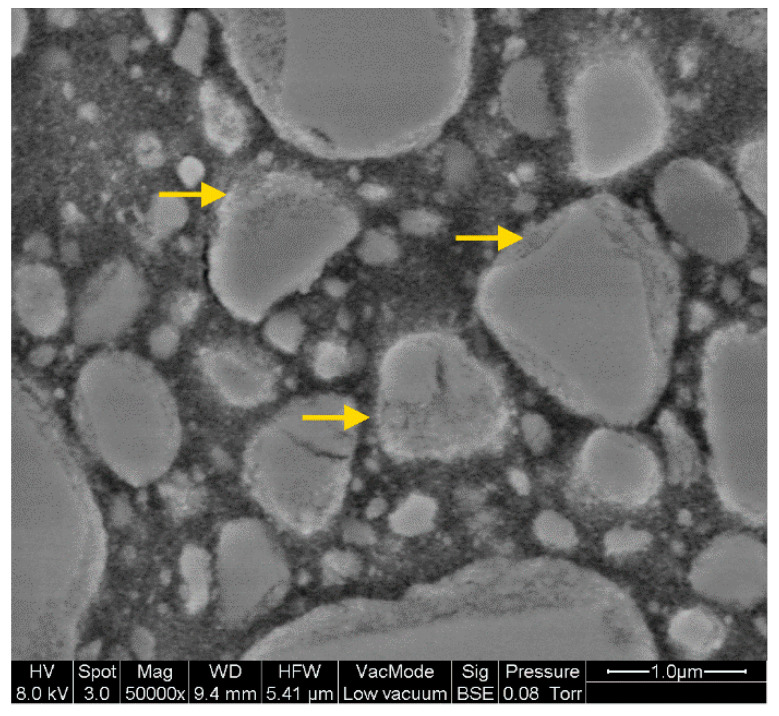
Example for porous edge area (yellow arrows) of filler particles in Lava Ultimate (3LU_A2LT).

The darker pixels in the SEM images and the lower silicon (Si) and higher carbon (C) contents in the edge areas identified in the fillers of 3LU (<7 µm) indicate a porous microstructure (Figure 9, yellow arrows). The manufacturer labelled the fillers as nanoclusters consisting of zirconia and silica with a size ranging between 0.6 and 1.4 µm. For the direct RBC, the pendant (Filtek Supreme, 3M) with an identical phase composition [40,41] cited in [42] reports agglomeration of nanoparticles that are partially calcined and infiltrated. Therefore, the porous microstructure identified in the current study might be caused by very small filler particles that stick to the larger particles because of their high surface tension. The higher porosity could lead to an improvement in the bonding behaviour between inorganic fillers and the organic resin matrix. In addition, the agglomeration of nanoparticles leads to multiple distinct fracture events, when the mechanical load is applied [42]. Mechanical stress caused by attrition, abrasion in combination with erosion, or grinding with a handpiece produces only partial loss of individual parts of the agglomerates rather than a larger particle filler.

EDS and XRD analyses showed that the filler fraction of all CAD/CAM RBCs investigated are Si-O-based X-ray amorphous phases which, depending on the manufacturer, often contain aluminium and barium (CBC, GCC, TC, and VGB) or zirconium (3LU and SB) as minor components. Barium and zirconium feature a high atomic mass, which is why even small amounts are sufficient to increase the radiopacity of the material dramatically [27]. The high atomic mass influences not only the X-ray opacity but also the linear absorption coefficient of visible light [43]. It is noticeable that manufacturers use either barium or zirconium, or in the case of LC, neither substance at all, to increase radiopacity. In the case of 3LU, the zirconium was detected in small crystals (large peaks width at half-height) and at SB in X-ray amorphous phase (probably glass phase) (Figure 5). When interpreting the chemical composition, it must be taken into account that only the microfillers and not nanofillers between them were analysed. Therefore, the composition may also differ locally [43,44].

The conventional viewpoint is that CAD/CAM RBCs feature a very homogeneous structure in comparison to their direct counterparts, which has been attributed to the industrial manufacturing process [6,28,45]. Nevertheless, we identified very large individual radiopaque fillers (max. length 224 µm in 3LU) and defects (pores and blowholes; max. length 340 µm in VGB) in some CAD/CAM RBCs in the current study (3LU, TC, and GCC). However, while the inhomogeneities themselves can be very large, their effects in relation to the total volume are still minor (<0.01 vol.%).

## 5. Conclusions

The null hypothesis of the current study cannot be confirmed. However, the following conclusions can be drawn:

1. All filler sizes are decisively larger than specified by the manufacturers. The filler distribution in all CAD/CAM RBCs investigated can be classified as midifill hybrid (equivalent diameter <11 µm).
2. The filler content in the CAD/CAM RBCs investigated tends to be larger than the values reported for direct RBCs [4].
3. With the exception of Lava Ultimate (3LU), which featured zirconium crystals with low crystallinity, all CAD/CAM RBCs showed only an X-ray amorphous phase stock.
4. In some CAD/CAM RBCs, inhomogeneities (X-ray opaque fillers or pores) with a size <340 µm were identified, but the local effects were minor in relation to the total volume (<0.01 vol.%).

## Figures and Tables

**Table 2:** Polishing protocol.

Paper ^1^	Suspension	Force	Rotation	Time
-	-	N	1/min	s
P220	distilled water	15	300	15
P500	distilled water	15	300	15
P1200	distilled water	15	300	15
P2000	distilled water	20	300	15
P4000	distilled water	20	300	15
MD DAC	DP Dac 3 µm	15	150	180
MD Nap	DP Nap ¼ µm	15	150	120
MD Nap	OP-S	15	150	120

^1^ P220, P500, P1200, P2000, and P4000 are silicon carbide papers.

**Table 3:** Inorganic content by weight (identified by thermogravimetric (TG) analyses) and volume (identified by SEM image analyses) in comparison to the specifications issued by the manufacturers.

		Unit	CBC	GCC	LC	SB	TC	VGB	3LU
**HT**	Volume	Vol.-%	46.6	37.2	-	71.2	44.9	51.8	67.1
	Weight	wt%	69.8	64.5	-	61.6	69.3	82.3	72.0
**LT**	Volume	Vol.-%	44.8	39.2	48.0	74.4	45.5	53.3	65.8
	Weight	wt%	69.4	64.8	68.8	62.5	69.9	83.1	72.3
**Manufacturer’s information**		Vol.-%	51.5	-	-	-	-	-	-
		wt%	70.7	-	70.0	-	71.1	86.0	80.0

**Table 4:** Properties of local discontinuities identified by µXCT in CAD/CAM RBCs (HT).

Density	Property	Unit	3LU	CBC	GCC	SB	TC	VGB
	Biggest	µm		53			77	340
**Low**		µm^3^		1.5 × 10^4^			1.4 × 10^4^	8.8 × 10^5^
	Total	Vol.-%		0.0032			0.0006	0.0970
	Biggest	µm	224		156		73	
**High**		µm^3^	1.6 × 10^6^		4.8 × 10^5^		1.4 × 10^5^	
	Total	Vol.-%	0.0602		0.0031		0.0057	

**Table 5:** Chemical composition of the filler particles (wt%. the CAD/CAM RBCs) analysed.

Elements	BRILLIANT Crios		Cerasmart		LuxaCAM Composite	Shofu Block HC		Tetric Cad		Grandio Blocs		Lava Ultimate	
	CBC		GCC		LC	SB		TC		VGB		3LU	
	LT	HT	LT	HT		LT	HT	MT	HT	LT	HT	HT	LT
C	16.1	15.2 17.2	15.8 15.9	16.4 16.0	13.3	14.9 8.5	13.5 7.8	14,4 14.0	14.4 14.0	7.2 8.8	7.7 7.8	12.4 11.2	7.5 10.3
O	48.8	49.1 49.4	44.3 45.1	45.5 45.7	53.3	54.8 56.1	52.6 55.9	49.0 49.7	49.0 49.7	51.2 50.6	50.4 51.1	51.3 53.2	53.0 53.4
Al	3.4	3.5 3.2	3.1 3.2	3.1 3.1	6.3	- -	-	3.6 3.6	3.6 3.6	4.1 4.1	4.2 4.1	-	-
Si	16.4	16.5 16.0	16.0 16.4	15.9 15.7	27.0	26.1 33.1	34.0 34.1	16.5 16.5	16.5 16.5	19.9 18.5	19.1 18.9	24.8 24.1	27.1 24.4
Ba	15.5	15.7 14.3	19.9 20.3	19.1 19.4	-	-	-	16.5 16.2	16.5 16.2	17.6 18.0	18.7 18.2	-	-
Zr	-	-	-	-	-	4.1 2.29	2.2 -	-	-	-	-	11.5 11.6	12.4 12.0

**Table 6:** Overview of characteristic parameters (top: manufacture data; bottom: own measured values).

Material	Code	Manufacturer	Filler Content		Composition ^1^	Granulometry		Inhomogeneity ^2^
			Mass	Volume		Size	Sphericity	
			wt%	vol.-%		µm	<1	± µm/vol.-%
BRILLIANT Crios	CBC	COLTENE Holding AG	70.7	-	Ba-glass Si-glass	<1 <0.02	-	-
			69.4–69.8	37.2–46.6	Si, Al, Br <X-r-a>	<3	0.58–0.63	−53/0.003
Cerasmart^TM^	GCS	GC Corporation	-	-	-	-	-	-
			64.5–64.8	37.2–39.2	Si, Al, Br <X-r-a>	<4	0.48–0.61	+156/0.003
Lava^TM^ Ultimate	3LU	3M Deutschland GmbH	≈80	-	Si-nano Zr-nano Zr-Si-cluster	<0.02 0.004–0.011 0.6–10	-	-
			72.0–72.3	65.8–67.1	Si, Zr Zirconia	<9	0.49–0.53	+222/0.060
Shofu Block HC	SB	Shofu Dental GmbH	-	-	-	-	-	-
			61.6–62.5	71.2–74.4	Si, Zr <X-r-a>	<11	0.71–0.83	/
Tetric^®^ CAD	TC	Ivoclar Vivadent GmbH	71.1	-	Si-Al-Br glass	<1 <0.02	-	-
			69.369.9	44.9–45.5	Si, Al, Br <X-r-a>	<4	0.51–0.53	+73/0.006 −77/0.001
Grandio^®^ blocs	VGB	VOCO GmbH	≈86	-	-	-	-	-
			82.3–83.1	51.8–53.3	Si, Al, Br <X-r-a>	<6	0.59–0.60	−340/0.097
LuxaCAM Composite	LC	DMG GmbH	≈70	-	Si-glass	-	-	-
			68.8	48.0	Si, Al <X-r-a>	<5	0.42	/

^1^ Top: main chemical elements; bottom: X-R-a = X-ray amorphous phases. ^2^ Longest inhomogeneity (in µm)/proportion of all inhomogeneities related to total volume (vol.-%).

## Data Availability

All data presented in this study are available in the article and as Supplementary Materials in a separate file.

## Supplementary Materials

The following are available online at https://www.mdpi.com/article/10.3390/ma14081986/s1. Table S1. Particle size and sphericity distribution. Table S2. Particle size and sphericity distribution. Table S3. Particle size and sphericity distribution. Table S4. TG analysis in detail. Figure S1. TG graphs of 3LU. Figure S2. TG graphs of CBC. Figure S3. TG graphs of GCC. Figure S4. TG graphs of LC. Figure S5. TG graphs of SB.; Figure S6. TG graphs of TC. Figure S7. TG graphs of VGB.
