# Correction: Koenig et al. Characterisation of the Filler Fraction in CAD/CAM Resin-Based Composites. *Materials* 2021, *14*, 1986

**DOI:** 10.3390/ma18051100

**Published:** 2025-02-28

**Authors:** Andreas Koenig, Julius Schmidtke, Leonie Schmohl, Sibylle Schneider-Feyrer, Martin Rosentritt, Hieronymus Hoelzig, Gert Kloess, Ketpat Vejjasilpa, Michaela Schulz-Siegmund, Florian Fuchs, Sebastian Hahnel

**Affiliations:** 1 Department of Dental Prosthetics and Materials Science, Leipzig University, 04103 Leipzig, Germany; 2 Department of Prosthetic Dentistry, Regensburg University Medical Centre, 93042 Regensburg, Germanymartin.rosentritt@klinik.uni-regensburg.de (M.R.); 3 Institute of Mineralogy, Crystallography and Materials Science, Leipzig University, 04275 Leipzig, Germany; 4 Institute of Pharmacy, Pharmaceutical Technology, Leipzig University, 04317 Leipzig, Germany; kv38gaxo@studserv.uni-leipzig.de (K.V.);

## Error in Figures

In the original publication [1], there was a mistake in Figures 2 and 3 as published: wrong unit (mm) in the x-axis. The corrected Figure 2 and Figure 3 with the correct unit (µm) in the *x*-axis appear below.

## Error in Table

In the top line of Table 4 was a mistake: Incorrect abbreviations 3LUA, CBCA, and SBA. The correct abbreviations in Table 4 are 3LU, CBC, and SB. The correct Table 4 appears below:

## Error in Supplementary Materials

In the original publication, there was a mistake in Tables S1–S3. The presented units of ferret diameter were incorrect and were revised from mm to µm.

The authors state that the scientific conclusions are unaffected. This correction was approved by the Academic Editor. The original publication has also been updated.

## Figures and Tables

**Figure 2:**
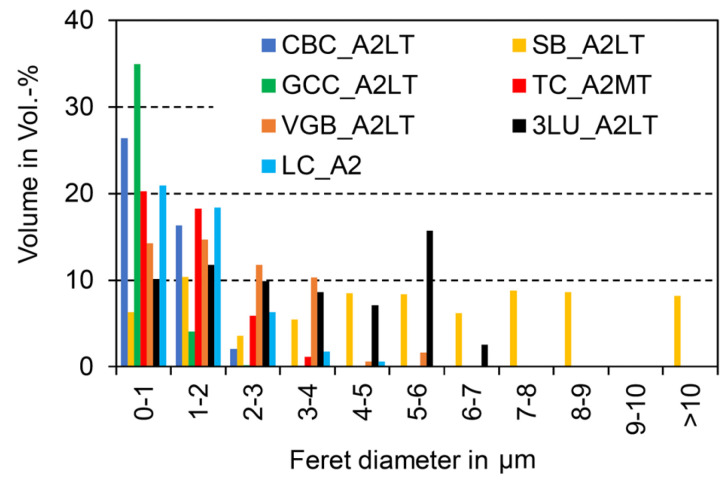
Filler size distribution (selection; see Supplementary Materials for overall results) based on one SEM picture with max. 5100 single particles.

**Figure 3:**
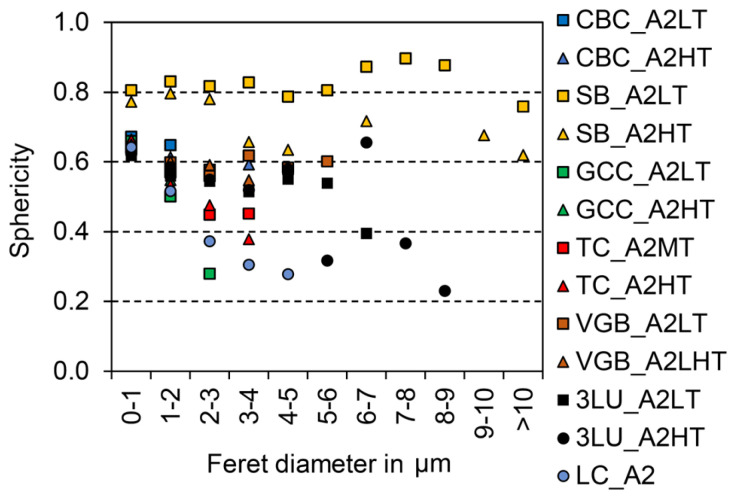
Filler sphericity distribution (1.0 indicates the cross section of the filler is a circle) based on one SEM picture with max. 5100 single particles.

**Table 4:** Properties of local discontinuities identified by µXCT in CAD/CAM RBCs (HT).

Density	Property	Unit	3LU	CBC	GCC	SB	TC	VGB
	Biggest	µm		53			77	340
**Low**		µm^3^		1.5 × 10^4^			1.4 × 10^4^	8.8 × 10^5^
	Total	Vol.-%		0.0032			0.0006	0.0970
	Biggest	µm	224		156		73	
**High**		µm^3^	1.6 × 10^6^		4.8 × 10^5^		1.4 × 10^5^	
	Total	Vol.-%	0.0602		0.0031		0.0057	
