# Supplementary material for: Characterisation of the Filler Fraction in CAD/CAM Resin-Based Composites
Source: Materials (Basel). 2021 Apr 15;14(8):1986. doi: 10.3390/ma14081986 (PMC8071413; doi:10.3390/ma14081986)
Supplement: Supplementary file 1 [file materials-14-01986-s001.zip › materials-1139143-supplementary.pdf]

materials

MDPI

Table S1. Particle size and sphericity distribution

Distribution	CBC_A2LT		CBC_A2HT		SB_A2LT		SB_A2HT	
Feret Diameter in µm	Area	Circ.	Area	Circ.	Area	Circ.	Area	Circ.
0-1	26.43	0.67	24.56	0.66	6.31	0.81	4.34	0.77
1-2	16.32	0.65	16.94	0.61	10.35	0.83	9.12	0.80
2-3	2.06	0.58	3.91	0.47	3.57	0.82	9.19	0.78
3-4	0.00	0.00	1.17	0.59	5.48	0.83	1.61	0.66
4-5	0.00	0.00	0.00	0.00	8.52	0.79	13.09	0.64
5-6	0.00	0.00	0.00	0.00	8.39	0.81	0.00	0.00
6-7	0.00	0.00	0.00	0.00	6.21	0.87	6.01	0.72
7-8	0.00	0.00	0.00	0.00	8.81	0.90	0.00	0.00
8-9	0.00	0.00	0.00	0.00	8.59	0.88	0.00	0.00
9-10	0.00	0.00	0.00	0.00	0.00	-1.00	7.77	0.68
>10	0.00	0.00	0.00	0.00	8.21	0.76	20.09	0.62
Total	44.80	-	46.58	-	74.44	-	71.22	-

Table S2. Particle size and sphericity distribution

Distribution	GCC_A2LT		GCC_A2HT		TC_A2MT		TC_A2HT	
Feret Diameter in µm	Area	Circ.	Area	Circ.	Area	Circ.	Area	Circ.
0-1	34.93	0.66	33.24	0.67	20.25	0.64	21.22	0.66
1-2	4.06	0.50	3.91	0.55	18.28	0.57	17.52	0.53
2-3	0.17	0.28	0.00	0.00	5.86	0.45	5.86	0.48
3-4	0.00	0.00	0.00	0.00	1.15	0.45	0.34	0.38
4-5	0.00	0.00	0.00	0.00	0.00	0.00	0.00	0.00
5-6	0.00	0.00	0.00	0.00	0.00	0.00	0.00	0.00
6-7	0.00	0.00	0.00	0.00	0.00	0.00	0.00	0.00
7-8	0.00	0.00	0.00	0.00	0.00	0.00	0.00	0.00
8-9	0.00	0.00	0.00	0.00	0.00	0.00	0.00	0.00
9-10	0.00	0.00	0.00	0.00	0.00	0.00	0.00	0.00
>10	0.00	0.00	0.00	0.00	0.00	0.00	0.00	0.00
Total	39.17	-	37.15	-	45.54	-	44.94	-

Table S3. Particle size and sphericity distribution.

Distribution	VGB_A2LT		VGB_A2HT		3LU_A2LT		3LU_A2HT		LC_A2	
Feret Diameter in µm	Area	Circ.	Area	Circ.	Area	Circ.	Area	Circ.	Area	Circ.
0-1	14.24	0.64	13.82	0.65	10.13	0.62	11.27	0.62	20.97	0.64
1-2	14.67	0.60	13.42	0.60	11.78	0.56	9.42	0.58	18.40	0.52
2-3	11.80	0.56	13.41	0.59	9.90	0.55	9.97	0.55	6.31	0.37
3-4	10.32	0.62	5.15	0.55	8.61	0.52	10.06	0.52	1.76	0.30
4-5	0.62	0.58	5.95	0.58	7.11	0.55	5.21	0.58	0.61	0.28
5-6	1.67	0.60	0.00	0.00	15.69	0.54	6.33	0.32	0.00	0.00
6-7	0.00	0.00	0.00	0.00	2.54	0.40	3.97	0.66	0.00	0.00
7-8	0.00	0.00	0.00	0.00	0.00	0.00	8.57	0.37	0.00	0.00
8-9	0.00	0.00	0.00	0.00	0.00	0.00	2.33	0.23	0.00	0.00
9-10	0.00	0.00	0.00	0.00	0.00	0.00	0.00	0.00	0.00	0.00
>10	0.00	0.00	0.00	0.00	0.00	0.00	0.00	0.00	0.00	0.00
Total	53.32	-	51.75	-	65.77	-	67.12	-	48.04	-

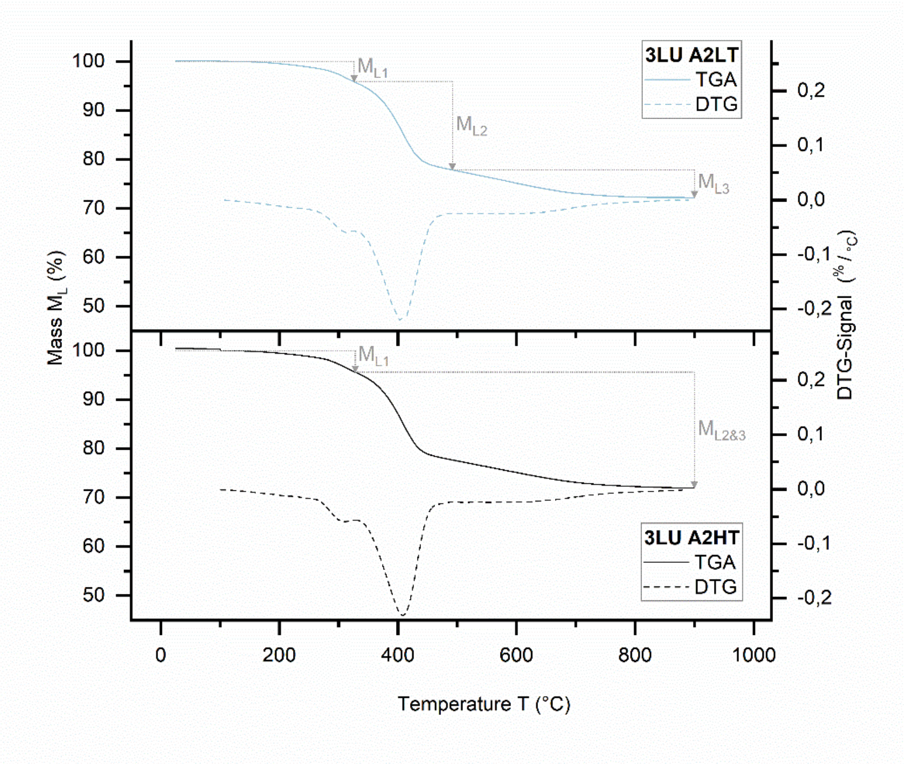

Figure S1. TG graphs of 3LU.

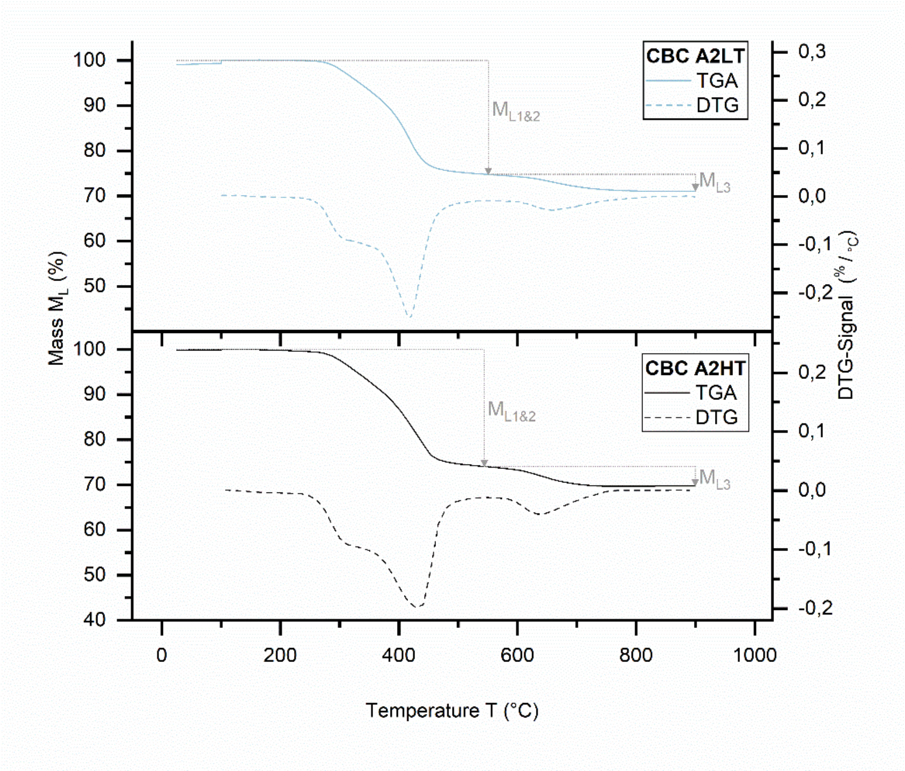

Figure S2. TG graphs of CBC.

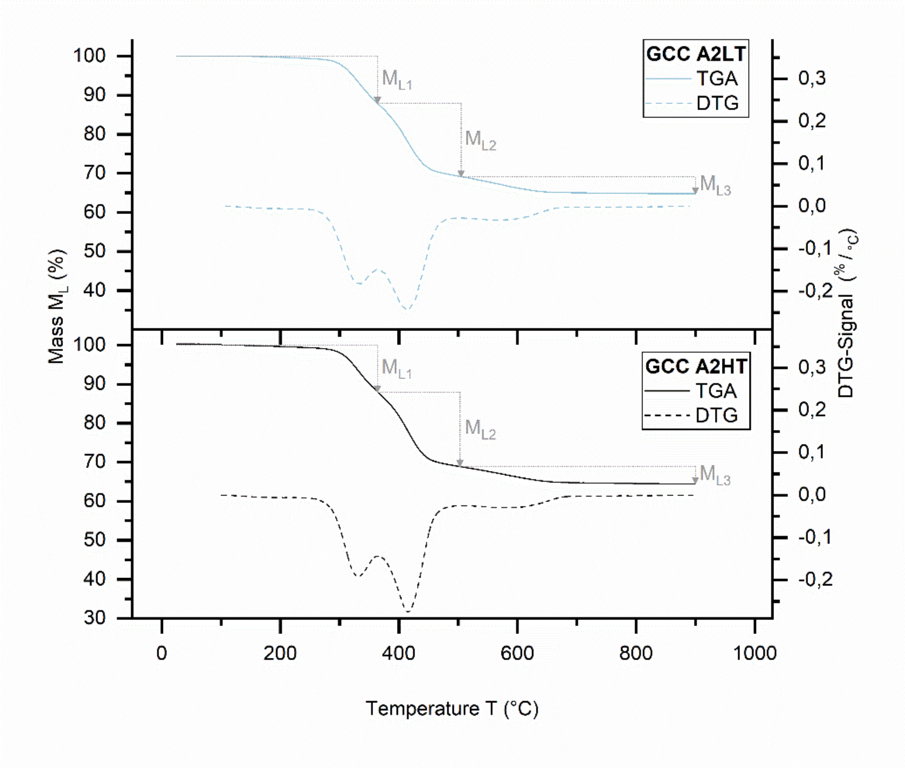

Figure S3. TG graphs of GCC.

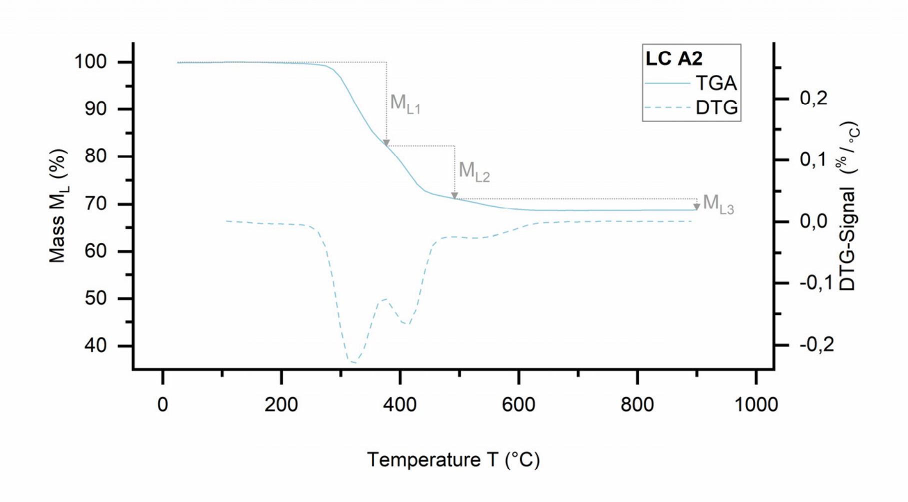

Figure S4. TG graphs of LC.

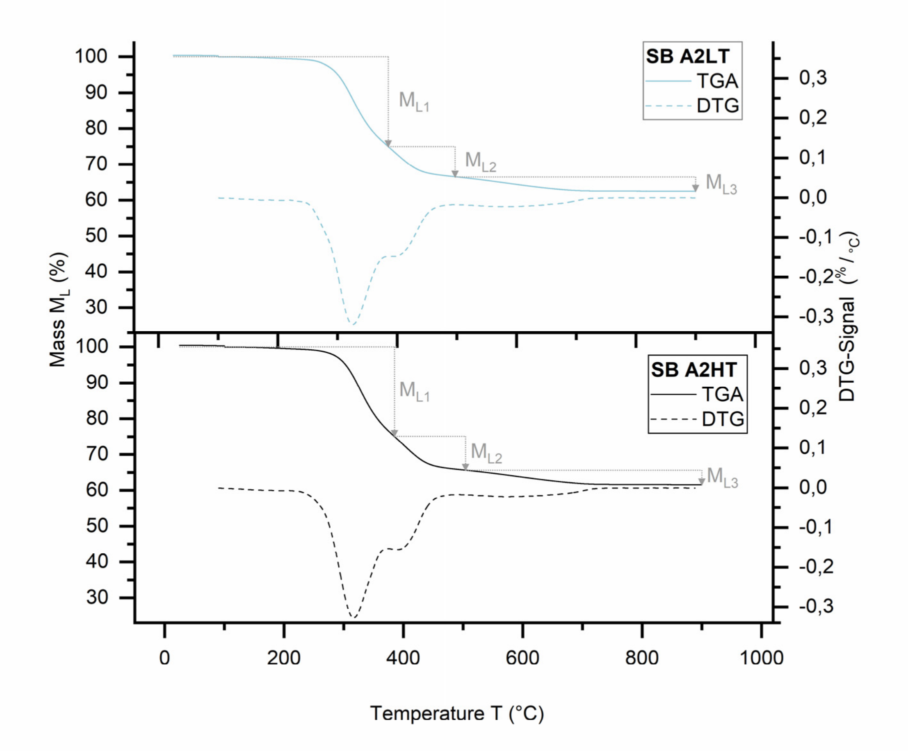

Figure S5. TG graphs of SB.

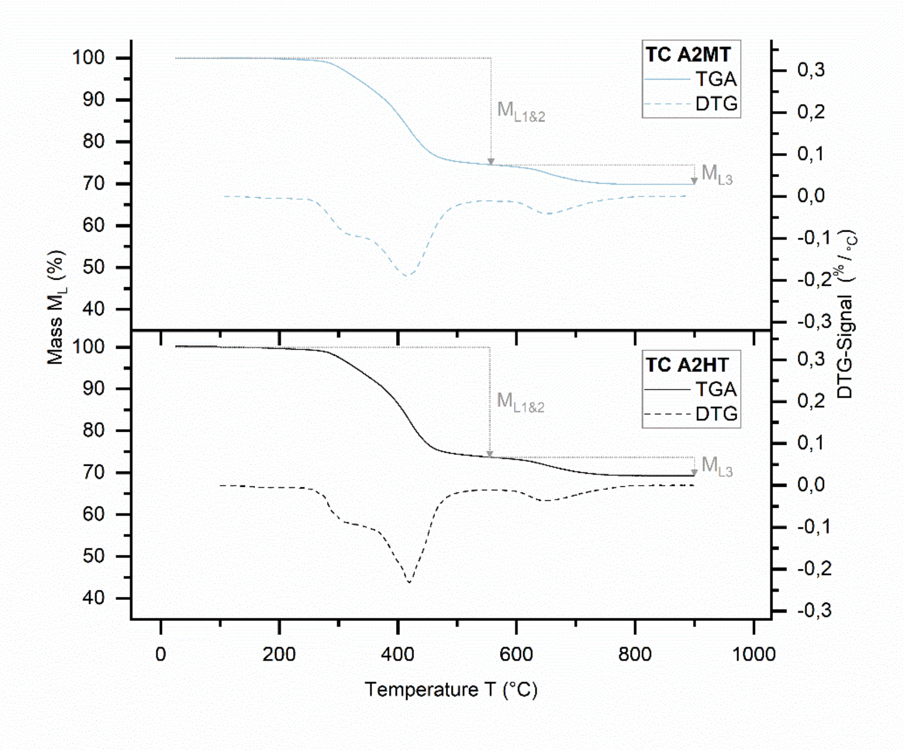

Figure S6. TG graphs of TC.

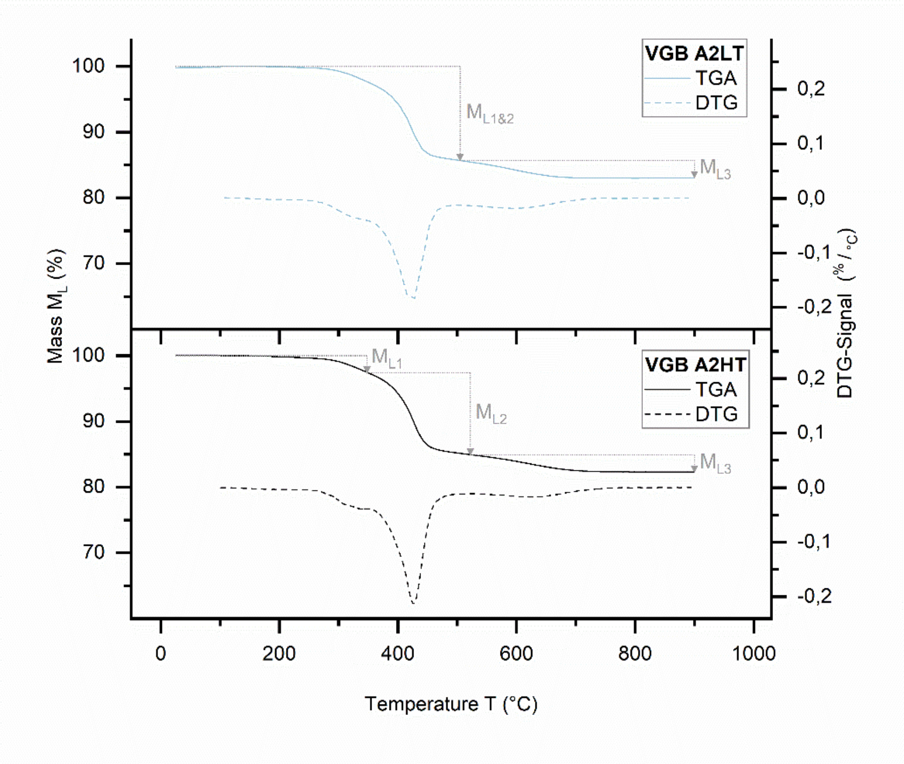

Figure S7. TG graphs of VGB.

Table S4. TG analysis in detail.

**3LU A2LT**	**Total**	**Step 1**	**Step 2**	**Step 3**	**Step 4**
Weight m_s in mg	13.247				
Start point T_A in °C	256	256	326	492	
End point T_B in °C	714	326	492	714	
Peak temperature T_P in °C	-	313	403	595	

**3LU A2HT**	**Total**	**Step 1**	**Step 2**	**Step 3**	**Step 4**
Weight m_s in mg	13.389				
Start point T_A in °C	255	255	328		
End point T_B in °C	732	328	732		
Peak temperature T_P in °C	-	312.5	407	563	
Mass loss M_L in %	28.0	4.4	23.6		

**VGB A2LT**	**Total**	**Step 1**	**Step 2**	**Step 3**	**Step 4**
Weight m_s in mg	13.439				
Start point T_A in °C	284		284	505	
End point T_B in °C	662		505	662	
Peak temperature T_P in °C		326	428	595	
Mass loss M_L in %	16.9		14.3	2.6	

**VGB A2HT**	**Total**	**Step 1**	**Step 2**	**Step 3**	**Step 4**
Weight m_s in mg	13.635				
Start point T_A in °C	283	283	348	522	
End point T_B in °C	692	348	522	692	
Peak temperature T_P in °C		340	426	629	
Mass loss M_L in %	17.7	2.6	12.5	2.6	

Table S4 continued.

TC A2MT	Total	Step 1	Step 2	Step 3	Step 4
Weight m_s in mg	14.280				
Start point T_A in °C	276		276	557	
End point T_B in °C	714		557	714	
Peak temperature T_P in °C		313	415	659	
Mass loss M_L in %	30.1		25.5	4.6	
**TC A2HT**	**Total**	**Step 1**	**Step 2**	**Step 3**	**Step 4**
Weight m_s in mg	13.797				
Start point T_A in °C	275		275	555	
End point T_B in °C	719		555	719	
Peak temperature T_P in °C		323	419	650	
Mass loss M_L in %	30.7		26.3	4.4	
**SB A2LT**	**Total**	**Step 1**	**Step 2**	**Step 3**	**Step 4**
Weight m_s in mg	12.651				
Start point T_A in °C	289	289	385	497	
End point T_B in °C	683	385	497	683	
Peak temperature T_P in °C		326	394	571	
Mass loss M_L in %	37.5	25.0	8.5	4.0	
**SB A2HT**	**Total**	**Step 1**	**Step 2**	**Step 3**	**Step 4**
Weight m_s in mg	13.686				
Start point T_A in °C	291	291	385	504	
End point T_B in °C	696	385	504	696	
Peak temperature T_P in °C		327	399	599	
Mass loss M_L in %	38.4	24.9	9.5	4.0	

Table S4 continued.

**LC A2**	**Total**	**Step 1**	**Step 2**	**Step 3**	**Step 4**
Weight m_s in mg	12.652				
Start point T_A in °C	286	286	377	492	
End point T_B in °C	591	377	492	591	
Peak temperature T_P in °C		326	415	518	
Mass loss M_L in %	31.2	17.7	11.1	2.4	
**GCC A2LT**	**Total**	**Step 1**	**Step 2**	**Step 3**	**Step 4**
Weight m_s in mg	13.305				
Start point T_A in °C	294	294	364	505	
End point T_B in °C	643	364	505	643	
Peak temperature T_P in °C		338	415	569	
Mass loss M_L in %	35.2	12.1	18.8	4.3	
**GCC A2HT**	**Total**	**Step 1**	**Step 2**	**Step 3**	**Step 4**
Weight m_s in mg	12.519				
Start point T_A in °C	297	297	365	503	
End point T_B in °C	657	365	503	657	
Peak temperature T_P in °C		331	416	579	
Mass loss M_L in %	35.5	12.1	19.0	4.4	
**CBC A2LT**	**Total**	**Step 1**	**Step 2**	**Step 3**	**Step 4**
Weight m_s in mg	12.943				
Start point T_A in °C	275		275	439	547
End point T_B in °C	704		439	547	704
Peak temperature T_P in °C		311	417	448	645
Mass loss M_L in %	30.6		21.0	5.3	4.3
**CBC A2HT**	**Total**	**Step 1**	**Step 2**	**Step 3**	**Step 4**
Weight m_s in mg	13.595				
Start point T_A in °C	275		275	544	
End point T_B in °C	694		544	694	
Peak temperature T_P in °C		313	428	634	
Mass loss M_L in %	30.2		25.9	4.3	
